# The Sch9 Kinase Regulates Conidium Size, Stress Responses, and Pathogenesis in *Fusarium graminearum*

**DOI:** 10.1371/journal.pone.0105811

**Published:** 2014-08-21

**Authors:** Daipeng Chen, Yang Wang, Xiaoying Zhou, Yulin Wang, Jin-Rong Xu

**Affiliations:** 1 State Key Laboratory of Crop Stress Biology for Arid Areas, College of Plant Protection, Northwest A&F University, Yangling, Shaanxi, China; 2 Department of Botany and Plant Pathology, Purdue University, West Lafayette, Indiana, United States of America; University of Nebraska-Lincoln, United States of America

## Abstract

Fusarium head blight caused by *Fusarium graminearum* is an important disease of wheat and barley worldwide. In a previous study on functional characterization of the *F. graminearum* kinome, one protein kinase gene important for virulence is orthologous to *SCH9* that is functionally related to the cAMP-PKA and TOR pathways in the budding yeast. In this study, we further characterized the functions of *FgSCH9* in *F. graminearum* and its ortholog in *Magnaporthe oryzae*. The Δ*Fgsch9* mutant was slightly reduced in growth rate but significantly reduced in conidiation, DON production, and virulence on wheat heads and corn silks. It had increased tolerance to elevated temperatures but became hypersensitive to oxidative, hyperosmotic, cell wall, and membrane stresses. The Δ*Fgsch9* deletion also had conidium morphology defects and produced smaller conidia. These results suggest that *FgSCH9* is important for stress responses, DON production, conidiogenesis, and pathogenesis in *F. graminearum*. In the rice blast fungus *Magnaporthe oryzae*, the Δ*Mosch9* mutant also was defective in conidiogenesis and pathogenesis. Interestingly, it also produced smaller conidia and appressoria. Taken together, our data indicate that the *SCH9* kinase gene may have a conserved role in regulating conidium size and plant infection in phytopathogenic ascomycetes.

## Introduction

*Fusarium graminearum* (teleomorph *Gibberella zeae*) is one of the major causal agents of wheat and barley head blight or scab in North America and other parts of the world [1], [2]. It also infects corn and other small grains. Like many other Fusarium species, *F. graminearum* produces several mycotoxins that are harmful to human and animals [3]. Deoxynivalenol (DON) is a trichothecene mycotoxin produced by this pathogen that is of major health concerns and monitored by many governments.

As a potent inhibitor of eukaryotic protein synthesis, DON is also a phytotoxin. In fact, the first virulence gene identified in *F. graminearum* is *TRI5* that encodes the trichodiene synthase essential for DON biosynthesis [4], [5]. Since the release of its genome sequence [6], molecular genetics and genomics studies of *F. graminearum* have advanced significantly. Expression profiling studies have identified a variety of genes with different expression levels during plant infection [7], [8]. Genes of various biochemical or biological functions have been identified as important virulence or pathogenicity factors in *F. graminearum*, including other genes involved in DON biosynthesis [9], [10], components of key signal transduction pathways [11], [12], [13], [14], transcription factors of different DNA-binding domains [15], [16], [17], and several enzymes required for primary metabolism [18], [19], [20]. Like many other filamentous ascomycetes, *F. graminearum* has three mitogen-activated protein (MAP) kinase pathways, and all of them play critical roles in pathogenesis and DON production [21], [22]. It also has the well-conserved cAMP signaling pathway that is involved in the switching from saprophytic growth to infectious growth [23]. Two genes encoding the catalytic subunits of PKA have distinct and overlapping functions in regulating developmental and plant infection processes in *F. graminearum* [24].

A total of 42 protein kinase genes were found to be important for plant infection by systemic characterization of the *F. graminearum* kinome [21]. One of them, FGSG_00472, is orthologous to *SCH9* of the budding yeast. The Sch9 protein kinase shares sequence similarity with the catalytic subunits of PKA and it is functionally related to cAMP signaling in response to nutrient availability in *Saccharomyces cerevisiae*. It inhibits PKA activity by regulating the localization of Tpk1/2/3 and stability of Tpk2 [25]. Disruption of *SCH9* increases PKA activities [25] and stress tolerance [26]. Similar to mutations in *RAS2* and *CYR1,* down-regulation of glucose signaling by deletion of *SCH9* increases longevity and resistance to oxidative stress and heat shock [27]. In *S. cerevisiae*, SCH9 also is a master regulator of protein synthesis. It is phosphorylated by TORC1 to regulate TORC1-dependent cellular processes, such as ribosome production and translation [28], [29]. The Sch9 kinase also is involved in the regulation of autophagy together with the TORC1 and cAMP-PKA pathways [30].

Although *SCH9* orthologs are well conserved in plant pathogenic fungi or filamentous ascomycetes, none of them have been functionally characterized. Considering the diverse functions of *SCH9* in *S. cerevisiae* and the importance of cAMP signaling in *F. graminearum*, in this study we further characterized the *Fgsch9* deletion mutant generated in the systemic characterization of the *F. graminearum* kinome [21]. Although it was only slightly reduced in growth rate, the Δ*Fgsch9* mutant was significantly reduced in DON production and virulence. It had increased tolerance to elevated temperatures but increased sensitivities to oxidative, hyperosmotic, cell wall, and membrane stresses. The Δ*Fgsch9* deletion mutant also was defective in conidiogenesis and produced smaller conidia. In the rice blast fungus *Magnaporthe oryzae*, the Δ*Mosch9* deletion mutant also was defective in conidiogenesis and pathogenesis. Interestingly, it also produced smaller conidia and appressoria. These results indicate that the *SCH9* kinase gene may have a conserved role in regulating conidium size and plant infection in plant pathogenic fungi.

## Results

### Deletion of the *FgSCH9* gene reduces hyphal growth and conidiation

In the functional study of the *F. graminearum* kinome, we noticed that the mutant deleted of FGSG_00472.3 was defective in plant infection [21]. This gene (named *FgSCH9*) encodes a serine/threonine protein kinase that is highly similar to yeast *SCH9*. It is well conserved in filamentous fungi and shares limited homology with MEK kinases of the MAP kinase pathways [31]. In this study, we first confirmed the *Fgsch9* mutant generated in an earlier study [21] by Southern blot analyses. When hybridized with a *FgSCH9* fragment amplified with primers F5 and R6, the wild type had the 4.4-kb *Eco*RI band but the Δ*Fgsch9* mutant SD1 had no hybridization signals. When probed with the *hph* fragment, PH-1 had no hybridization signals. Mutant SD1 had a 2.8-kb band, which is the expected size derived from the gene replacement event (Fig. S1).

**Figure 1:**
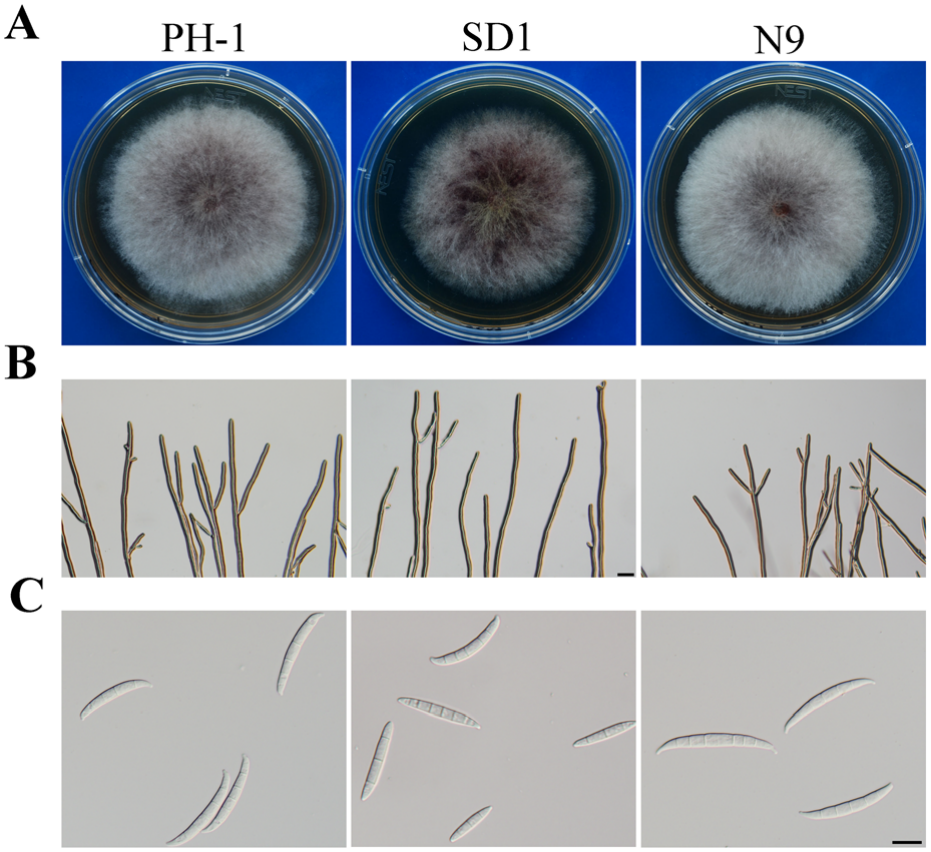
Colony morphology, hyphal growth and conidia morphology. **A.** Colony morphology of the wild type PH-1, Δ*Fgsch9* mutant SD1, and Δ*Fgsch9*/*SCH9* transformant N9 cultures grown on PDA. Photographs were taken after incubation for 3 days. **B.** Hyphae of PH-1, SD1, and N9 cultured on 1/2 CM slab agar for 36 h. **C.** Conidia morphology of PH-1, N9, and SD1. Bar = 10 µm.

The Δ*Fgsch9* mutant was reduced in growth rate and conidiation (Table 1). On PDA agar plates, mutant SD1 produced less aerial hyphae than PH-1 (Fig. 1A). We also noticed that mutant SD1 was reduced in hyphal branching at the edges of colonies formed on 1/2 CM for 36 h in comparison with PH-1 (Fig. 1B). In addition, 70.4±7.9% of the Δ*Fgsch9* conidia had morphological defects. Instead of forming typical Fusarium conidia, the Δ*Fgsch9* mutant produced conidia that lacked foot cells or had fewer septa (Fig. 1C). After measuring 80 conidia per replicate, the average size of the wild-type conidia was estimated to be 47.3±8.3×5.3±0.7 µm (length×width) with measurements from three independent experiments. In the Δ*Fgsch9* mutant strain SD1, conidia had the average size of 38.0±6.1×5.2±0.6 µm, which is approximately 20% smaller than the wild-type conidia. On average, the wild-type conidia had 4.3±0.6 septa after measuring 150 conidia per replicate and repeating three times. The *Fgsch9* mutant produced conidia with 3.1±0.5 septa per conidium.

**Table 1:** Phenotypes of the *Fgsch9* mutants in growth, conidiation, pathogenesis, and DON production.

Strain	Growth rate (mm/d)^a^	Conidiation (×10^6^/ml)^b^	Disease Index^c^	DON (ppm)^d^
PH-1 (WT)	18.9±0.1^A^ *	3.0±0.2^A^	14.7±1.5^A^	1611.5±92.6^A^
SD1 (▵*Fgsch9*)	16.2±0.2^B^	1.3±0.3^B^	5.6±1.0^B^	571.1±109.4^B^
SE1	18.5±0.1^A^	2.7±0.2^A^	13.2±0.7^A^	1525.2±86.3^A^
N9 (▵*Fgsch9/FgSCH9*)	18.0±0.2^A^	2.4±0.4^A^	14.5±0.6^A^	1593.4±95.9^A^

a Daily extension in colony radius on PDA plates.

b Conidiation in CMC cultures incubated at 25°C for 5 days.

c Diseased spikelets per wheat head at 14 dpi.

d DON production in infected wheat kernels harvested from inoculated wheat heads 14 dpi.

*Mean and standard deviation were calculated from four independent replicates. Data were analyzed with the protected Fisher’s Least Significant Difference (LSD) test. Different letters were used to mark statistically significant difference (P = 0.05).

### The Δ*Fgsch9* mutant has increased tolerance to elevated temperatures

**Figure 2:**
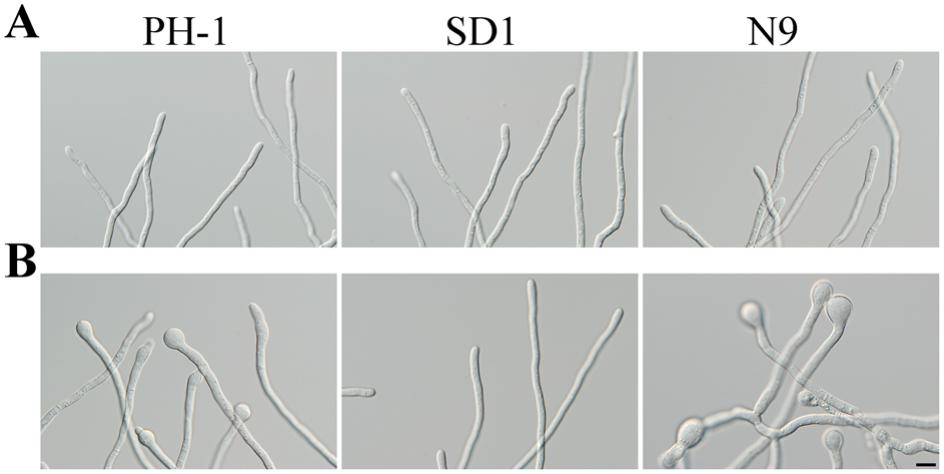
Increased tolerance to elevated temperatures in germ tubes of the Δ*Fgsch9* mutant. Conidia of the wild type PH-1, Δ*Fgsch9* mutant SD1, and Δ*Fgsch9*/*SCH9* transformant N9 were inoculate in YEPD at 25°C (**A**) and 30°C (**B**). Photographs were taken after incubation for 18 h. Bar = 10 µm.

To test whether the Δ*Fgsch9* mutant has increased tolerance to elevated temperatures, we assayed germ tube growth after incubation in YEPD for 18 h at 30°C. Similar to incubation at 25°C (Fig. 2A), both PH-1 and Δ*Fgsch9* mutant SD1 germinated and produced germ tubes at 30°C. However, germ tubes of PH-1 often had apical and intercalary swollen bodies when incubated at 30°C but not at 25°C (Fig. 2B). Germ tube growth was normal in the Δ*Fgsch9* mutant SD1 at either 25 or 30°C. These observations suggest that deletion of *FgSCH9* increased tolerance to elevated temperatures, which is similar to what was observed in *S. cerevisiae* [26].

### The Δ*Fgsch9* mutant is defective in responses to various environmental stresses

**Figure 3:**
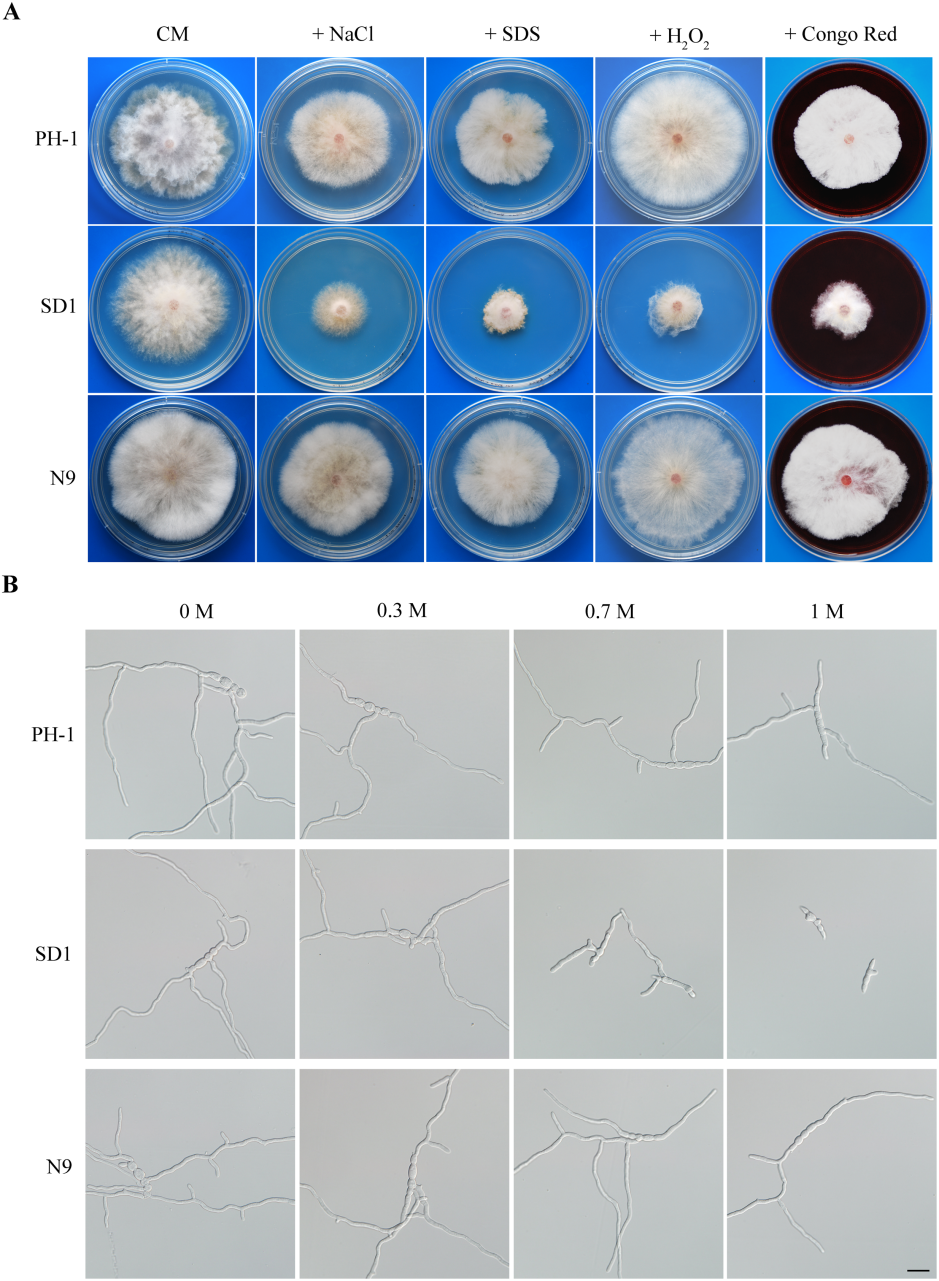
Defects of the Δ*Fgsch9* mutant in response to oxidative, membrane, hyperosmotic, and cell wall stresses. **A.** Colonies of PH-1, Δ*Fgsch9* (SD1), and complemented transformant N9 cultured on CM plates with 0.05% H_2_O_2_, 0.7 M NaCl, 0.01% SDS, or 300 µg/ml Congo Red at 25°C for three days. **B.** Conidium germination of PH-1 SD1 and N9 with different concentrantion of NaCl for 12 h. Bar = 20 µm.

To test whether deletion of *FgSCH9* affects responses to other stresses, we assayed colonial growth of the Δ*Fgsch9* mutant on CM plates with four different chemicals that are representative of oxidative, cell wall, hyperosmotic, and membrane stresses. In comparison with the wild type, mutant SD1 produced significantly smaller colonies after incubation for 3 days in the presence of 0.05% H_2_O_2_, 300 µg/ml Congo red, 0.7 M NaCl, or 0.01% SDS (Fig. 3A). The Δ*Fgsch9* mutant had restricted hyphal growth, particularly on CM with 0.01% SDS. These results indicated that the Δ*Fgsch9* mutant had increased sensitivities to oxidative, membrane, hyperosmotic, and cell wall stresses. *FgSCH9* must be involved in responses to general environmental stresses in *F. graminearum*. In the budding yeast, the Δ*Fgsch9* mutant has increased sensitivities to oxidative and osmotic stresses [26], [32].

To confirm its defects in response to hyperosmotic stress, we assayed conidium germination and germ tube growth in the Δ*Fgsch9* mutant in YEPD with different concentrations of NaCl. After incubation at 25°C for 12 h, germ tube growth was similar between mutant SD1 and PH-1 in the presence of 0.3 M NaCl (Fig. 3B). However, although germination was not inhibited, germ tube growth was significantly reduced by 0.7 M or 1 M NaCl in mutant SD1 but not in PH-1 (Fig. 3B). Therefore, germ tube growth, similar to hyphal growth, is sensitive to hyperosmotic stress in the Δ*Fgsch9* mutant.

### New hyphal growth is observed inside dead hyphae of the Δ*Fgsch9* mutant

**Figure 4:**
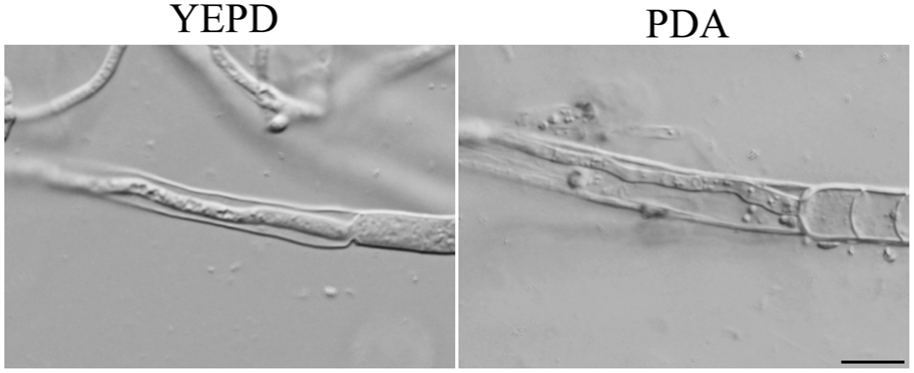
New hyphal growth inside dead compartments in the Δ*Fgsch9* mutant. Hyphae of the Δ*Fgsch9* mutant SD1 grown on YEPD or PDA slab agars were examined by phase contrast microscopy. Bar = 10 µm.

Interestingly, old hyphae of the Δ*Fgsch9* mutant often had empty, dead intercalary compartments, which may be related to its defects in cell wall integrity. In hyphae from YEPD or PDA cultures, we were able to observe new hyphal growth inside the empty compartments (Fig. 4). Close examination indicated that new hyphal growth emerged through the septal pore area from flanking compartments that were still alive (Fig. 4). These observations indicate that the Δ*Fgsch9* mutant must be able to plug up the septal pore when some hyphal compartments were damaged or became empty. However, this plug up is incomplete or reversible and the Δ*Fgsch9* mutant can re-establish polarized growth through the plugged septal pore area.

### The Δ*Fgsch9* mutant is defective in plant infection

To determine the role of *FgSCH9* in pathogenesis, we inoculated flowering wheat heads with conidia from PH-1 and mutant SD1. The spikelets of susceptible wheat cultivar Xiaoyan22 drop-inoculated with the Δ*Fgsch9* mutant still developed typical wheat scab symptoms. However, mutant SD1 was defective in spreading from the inoculated kernels to other spikelets on the same wheat heads. At 14 days post-inoculation (dpi), the average disease index (diseased spikelets per head) was 5.6 for mutant SD1 and 14.7 for PH-1 (Table 1), which was approximately a 62% reduction in virulence. Because it is an important virulence factor [4], [5], we also assayed DON production in diseased wheat kernels harvested at 14 dpi. In comparison with PH-1, the Δ*Fgsch9* mutant also was reduced in DON production (Table 1).

**Figure 5:**
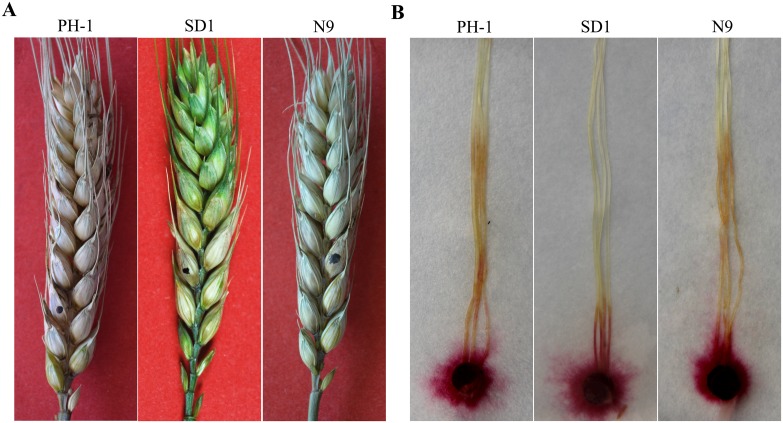
Infection assays with flowering wheat heads and corn silks. **A.** Flowering wheat heads were drop-inoculated with conidia from the wild type PH-1, Δ*Fgsch9* mutant SD1, and complemented strain N9. Typical wheat heads were photographed 14 dpi. The inoculated kernel was marked with a black dot. **B.** Corn silks were inoculated with culture blocks of the same set of strains and incubated at 25°C. Symptoms were observed at 5 dpi.

*F. graminearum* also is a corn pathogen and it can cause Gibberella ear rot and stalk rot. In infection assays with corn silks, both PH-1 and mutant SD1 were able to colonize and cause discoloration. However, the extent of discoloration was significantly reduced in the Δ*Fgsch9* mutant in comparison with the wild type (Fig. 5B). On corn silks inoculated with mutant SD1, discoloration was restricted to the inoculation site. Based on data from three independent experiments, the average discolored region on corn silks inoculated with PH-1 and the *Fgsch9* mutant was 47.6±4.8 mm and 16.7±3.7 mm, respectively. These results further indicate that *FgSCH9* is an important virulence factor in *F. graminearum*.

### Expression of *FgSCH9*-GFP fully complemented the defects of mutant SD1

For complementation assays, we generated the *FgSCH9-*GFP fusion construct under its own promoter control and introduced it into the Δ*Fgsch9* mutant SD1. Strain N9 was one of the resulting Δ*Fgsch9/FgSCH9-*GFP transformants identified by PCR assays. It was normal in growth rate, conidiation, and conidium morphology (Fig. 1; Table 1). The defects of mutant SD1 in stress responses and plant infection also were rescued in transformant N9 (Fig. 3; Fig. 5). These results indicate that deletion of *FgSCH9* is directly responsible for all the phenotypes of the Δ*Fgsch9* mutant.

**Figure 6:**
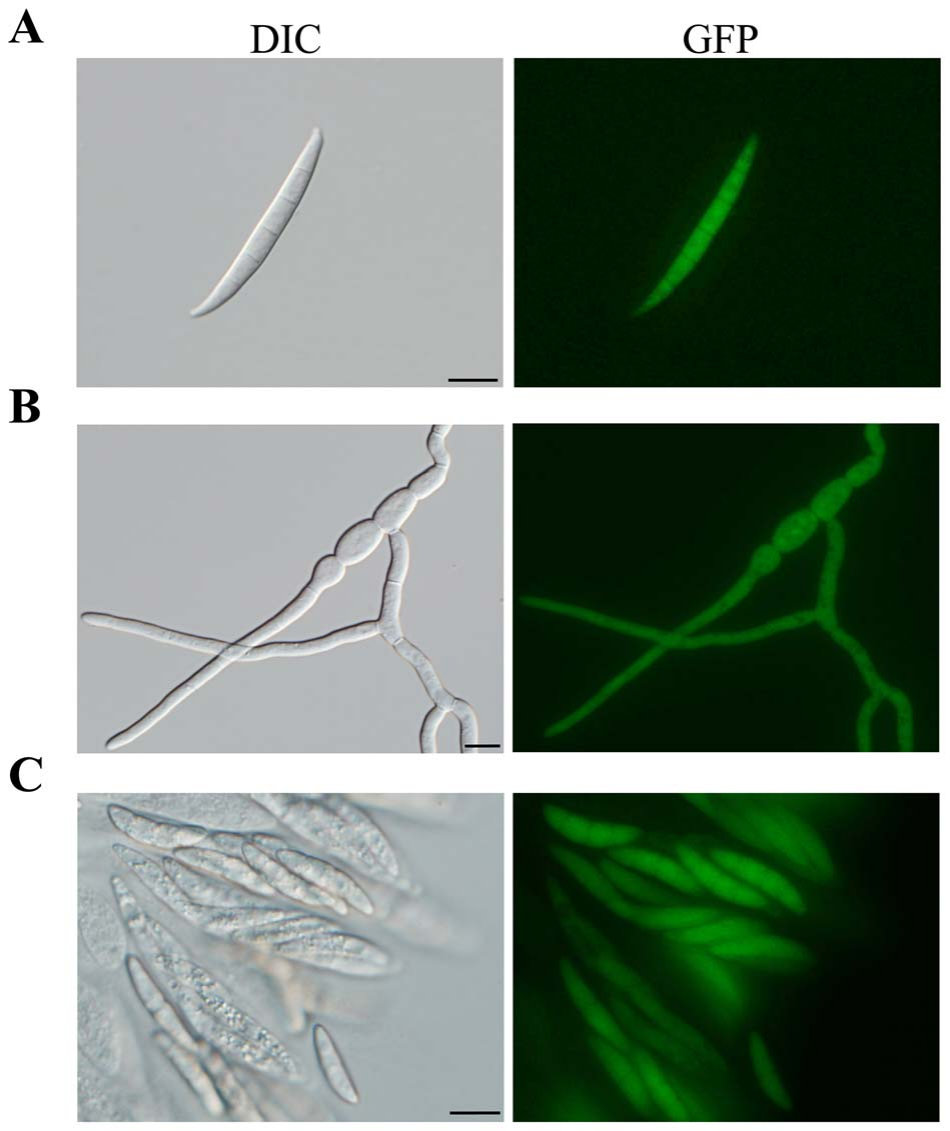
Subcellular localization of FgSch9-GFP fusion proteins. Freshly harvested conidia (**A**), 12 h germlings (**B**), and ascospores (**C**) of the Δ*Fgsch9*/*FgSCH9*-GFP transformant N9 were examined by phase contrast (DIC) or epifluorescence (GFP) microscopy. GFP signals were present mainly in the cytoplasm. Bar = 10 µm.

To determine the expression and subcellular localization of FgSCH9 in different growth and developmental stages, we examined GFP signals in transformant N9 by epifluorescence microscopy. In conidia and germ tubes, GFP signals of similar strength were observed in the cytoplasm (Fig. 6). Similar results were obtained in hyphae and ascospores. These data indicate that *FgSCH9* was expressed in all the cell types examined and it had no distinct subcellular localization pattern in *F. graminearum*, which is similar to the localization of Sch9 in *S. cerevisiae* [33].

### The *Mosch9* mutant also was reduced in conidium size and virulence

**Figure 7:**
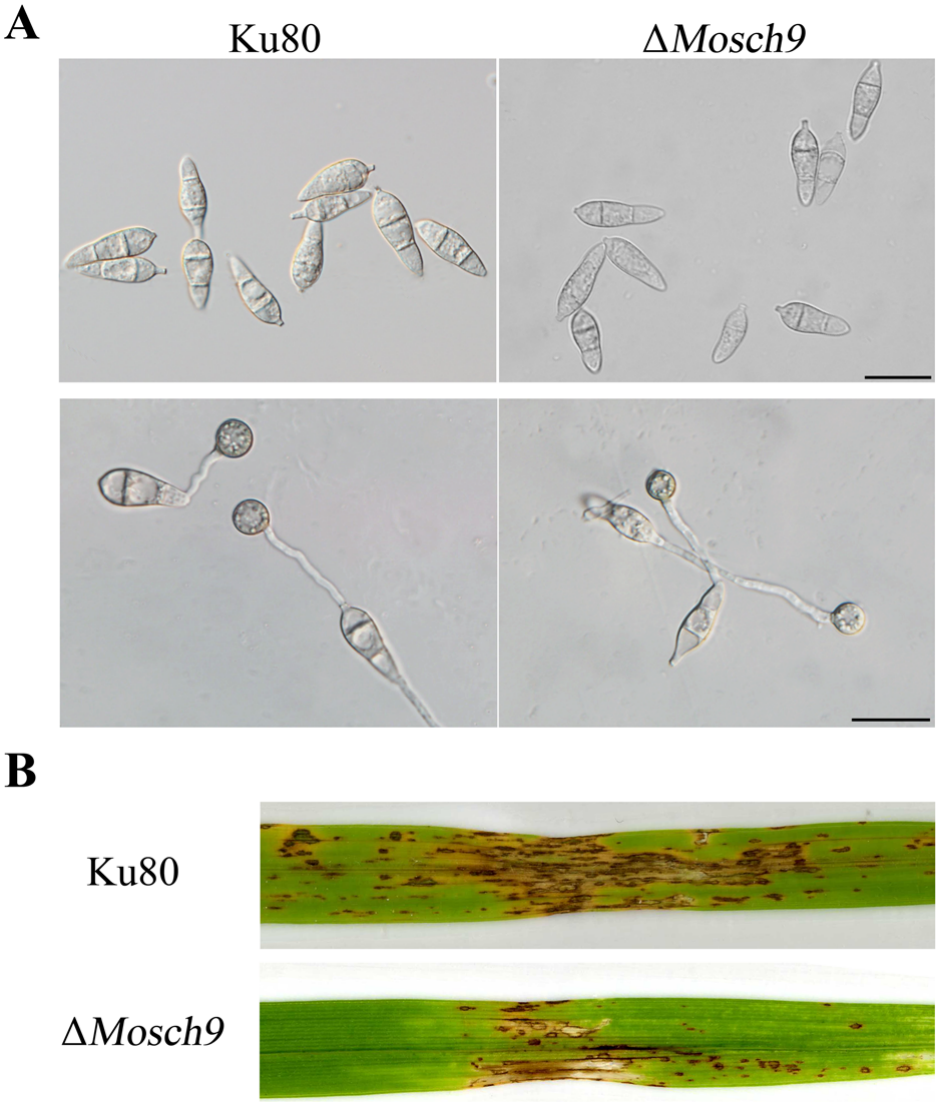
Phenotypes of the Δ*Mosch9* mutant in conidia, appressoria, and pathogenesis. **A.** Appressorium formation assays with conidia of the wild type Ku80 and the Δ*Mosch9* mutant on hydrophobic plastic coverslips. Typical samples were photographed after incubation at 25°C for 24 h. **B.** Rice leaves wound-inoculated with Ku80 and the Δ*Mosch9* mutant. Lesion formation was observed 7 dpi.

To verify the function of *FgSCH9* in controlling cell size and virulence, we identified its ortholog in the rice blast fungus *M. oryzae*, MGG_14773.6 that was named *MoSCH9* in this study. The *MoSCH9* gene replacement construct was generated by the ligation-PCR approach [34] and transformed into protoplasts of strain Ku80 [35]. The Δ*Mosch9* deletion mutant also was reduced in conidium size (Fig. 7A). Whereas the average size of wild-type conidia was 18.1±1.5×6.0±0.7 µm (length×width), conidia of the Δ*Mosch9* mutant were 15.5±1.7×5.1±0.6 µm. Interestingly, appressoria formed by the Δ*Mosch9* mutant on hydrophobic surfaces also were smaller than those of strain Ku80 (Fig. 7A). On average, the diameter of mutant appressoria was 6.9±0.4 µm. The average size of wild-type appressoria was 8.4±0.5 µm. In infection assays with rice leaves, the Δ*Mosch9* mutant also was reduced in virulence (Fig. 7B). In comparison with Ku80, the mutant produced fewer lesions (Fig. 7B). These results indicate that the *SCH9* orthologs may have conserved functions in regulating cell size and plant infection in fungal pathogens.

## Discussion

In the budding yeast, the Sch9 kinase is functionally related to the cAMP-signaling and TORC1 pathways [25], [28], [30]. These two well-conserved pathways recently were shown to be involved in various development and infection processes in *F. graminearum* [23], [24], [36]. Although Sch9 orthologs are well conserved in filamentous ascomycetes, none of them have been functionally characterized. In this study, we found that the Δ*Fgsch9* mutant had a reduced growth rate and produced smaller conidia, which is similar to the *sch9* deletion mutant of *S. cerevisiae* that exhibits slow growth and small cell size [33], [37], [38], [39]. In *M. oryzae*, conidia of the Δ*Mosch9* mutant also were smaller than those of the wild type. Even the appressoria produced by the Δ*Mosch9* mutant were smaller. In *C. albicans*, the cell size of the *CaSCH9* deletion mutant was significantly reduced in comparison with that of the wild type strain [40]. Therefore, *SCH9* orthologs may have a conserved function in regulating conidium size in filamentous ascomycetes. In *S. cerevisiae*, *SCH9* and *SFP1* are required for carbon-source modulation of cell size [33].

The TOR complex is well-conserved from yeast to human and it is involved in regulating growth-related signaling. It controls growth in response to nutrients by regulating translation, transcription, ribosome biogenesis, nutrient transport, and autophagy [29], [30], [41], [42]. In *S. cerevisiae*, *SCH9* functions downstream from the TOR pathways for properly regulating ribosome biogenesis, translation initiation, and entry into G_0_ phase [28]. In *F. graminearum*, the TOR pathway plays critical roles in regulating vegetative differentiation and virulence [36]. It is likely that *FgSCH9* is also functionally related to the TOR pathway in *F. graminearum*. Unlike *S. cerevisiae* that has *TOR1* and *TOR2*, two TOR kinase genes, *F. graminearum* has a single essential TOR kinase gene [21]. It will be interesting to determine the functional relationship between FgTor1 and FgSch9 by assaying the phosphorylation level or activity of FgSch9 in rapamycin-treated samples.

In the budding yeast, the Sch9 kinase is functionally related to the Ras-cAMP signaling pathway and it shares a large number of phosphorylation targets with PKA, such as Hog1 and Pfk2 [43]. Sch9 inhibits PKA activity and disruption of *SCH9* increases PKA activities [25]. In the fission yeast *Schizosaccharomyces pombe*, Sck1 and Sck2 are homologous to Sch9. Overexpression of the *sck1* or *sck2* gene can suppress the loss of PKA activity [44], [45]. *F. graminearum* has only one Sch9 ortholog but two genes encoding catalytic subunits of PKA. Whereas deletion of *CPK2* had no detectable phenotype, the *cpk1* mutant was reduced in growth, virulence, DON production, and conidiogenesis [24]. The *cpk1 cpk2* double mutant had more severe defects in growth and was non-pathogenic [24]. In addition, Sch9 and PKA control parallel pathways that converge on the Rim15 kinase in *S. cerevisiae* [41], [46]. In *M. oryzae,* the *rim15* deletion mutant has a slower growth rate, slightly increased sensitivity to hyperosmotic stress, and reduced hyphal melanization and virulence [47]. In *F. graminearum*, the *Fgrim15* mutant was reduced in conidiation and virulence [21].

In *S. cerevisiae*, *SCH9* also is involved in the signal transduction facilitator function of the Hsp90 chaperone complex that is required for the maturation of hundreds of diverse client proteins [48]. The *sch9* mutant has increased stress resistance [26]. In human pathogen *Cryptococcus neoformans*, the *sch9* deletion mutant also had increased thermal tolerance [49]. In *F. graminearum*, the Δ*Fgsch9* mutant had increased tolerance to elevated temperatures during germ tube growth. Germ tubes of the Δ*Fgsch9* mutant were normal but the wild type produced apical and intercalary swollen bodies when incubated at 30°C.

In *F. graminearum*, the Δ*Fgsch9* mutant had increased sensitivity to 0.7 M NaCl, suggesting that *FgSCH9* is important for response to hyperosmotic stress. In the budding yeast, Sch9 is involved in response to hyperosmotic stress via its association with Hog1 and co-regulation of subsets of genes such as *GRE2* and *CTT1* [32]. Nevertheless, the Δ*Fgsch9* mutant had increased sensitivity to SDS and Congo red as well. In addition, we found that the Δ*Fgsch9* mutant had the hyphae-in-hyphae phenotype and increased tolerance to elevated temperatures. Therefore, it is likely that *FgSCH9* is required for general stress responses in *F. graminearum*.

Unlike the *cpk1 cpk2* double mutant [24], the Δ*Fgsch9* mutant was still pathogenic. However, it was significantly reduced in virulence on flowering wheat heads and corn silks. Although *SCH9* orthologs are well-conserved in plant pathogenic fungi or filamentous ascomyctes, none of them have been characterized. However, its ortholog is known to be important for virulence in human pathogens *C. albicans* and *C. neoformans*. The *CaSCH9* deletion mutant was attenuated in virulence in a mouse mode of systemic candidiasis due to its defects in yeast growth and filamentation [40]. In *C. neoformans*, the *SCH9* ortholog functions both independently of and in conjunction with the cAMP-PKA pathway in pathogenesis [49]. In *F. graminearum*, DON is an important virulence factor and the Δ*Fgsch9* mutant was significantly reduced in DON production in infected wheat kernels. In addition, the Δ*Fgsch9* mutant had a reduced growth rate and increased sensitivity to oxidative and other stresses. All these factors may contribute to the defects of the Δ*Fgsch9* mutant in plant infection.

The Δ*Fgsch9* mutant had increased sensitivity to cell wall stresses, indicating defects in cell wall integrity. However, unlike the *mgv1* mutant [22], deletion of *FgSCH9* had no effect on hyphal fusion in *F. graminearum*. Interestingly, old hyphae of the Δ*Fgsch9* mutant often had empty, dead intercalary compartments, likely due to its defects in cell wall integrity. In some of them, new, narrow hyphae were produced from the middle of septa and grew into the empty, dead old hyphae. It appears that the Δ*Fgsch9* mutant can plug up the septal pore when hyphal compartments become damaged. However, it is defective in this process and somehow can regain polarized growth through the plugged septal pore areas. To our knowledge, this phenomenon of new hyphal growth inside old hyphal compartments has not been reported in *F. graminearum*. Recently, we found that the *tub2* deletion mutant also had similar defects (Zhang and Xu, unpublished). It will be interesting to determine the molecular mechanisms involved in the regulation of septal pore plugging and further growth inside empty hyphal fragments or re-establishment of hyphal tip growth at the septal pore.

## Materials and Methods

### Strains and culture conditions

The wild-type strain PH-1 and all the transformants of *F. graminearum* generated in this study were routinely cultured on PDA agar plates [50]. Growth rate and conidiation were assayed as described [24], [51]. DNA was extracted from vegetative hyphae harvested from liquid YEPD (1% yeast extract, 2% peptone, 2% glucose). Protoplast preparation and PEG-mediated transformation were performed as described [22]. Hygromycin B (Calbiochem, La Jolla, CA) and geneticin (Sigma, St. Louis, MO) were added to the final concentrations of 300 and 350 µg/ml, respectively, to the TB3 medium (0.3% yeast extract, 0.3% casamino acids, 3% glucose) for transformant selection. To test sensitivity against various stresses, vegetative growth was assayed on CM plates with 0.05% H_2_O_2_, 0.01% SDS, 300 µg/ml Congo Red, or 0.7 M NaCl as described [21], [52]. For assaying conidium germination and germ tube growth, conidia were shaken in YEPD (5×10^4^ conidia/ml) at 175 rpm for 12 h before examination.

The *M. oryzae* strain Ku80 [35] and transformants generated in this study were cultured on oatmeal agar plates (OTA) as described [53]. Transformants were selected on TB3 with 300 µg/ml zeocin (Invitrogen, Carlsbad, CA) or 250 µg/ml hygromycin B [54].

### Generation of *FgSCH9*-GFP fusion constructs and complementation strains

To generate the *SCH9*-GFP fusion constructs, PCR products containing the genomic fragments of the target gene and its promoter sequence were amplified with primers GFPNAT/F and GFPNAT/R (Table S1) and cloned into pFL2 by the yeast gap repair approach [55], [56]. The *FgSCH9*-GFP fusion construct was confirmed by sequencing analysis and transformed into protoplasts of the Δ*Fgsch9* mutant. Geneticin-resistant transformants harboring the transforming *FgSCH9*-GFP construct were identified by PCR and confirmed by the presence of GFP signals. The Δ*Fgsch9*/*gSCH9*-GFP complemented transformant N9 was selected for phenotype analysis and subcellular localization analysis of GFP signals.

### Infection with *F. graminearum* and DON production assays

For infection assays with flowering wheat heads of cultivars XiaoYan 22 or Norm, conidia were harvested from 5-day-old CMC cultures and re-suspended in sterile distilled water to 2.0×10^5^ conidia/ml [57], [58]. The fifth spikelet from the base of the spike was inoculated with 10 µl of conidium suspensions as described [59]. Inoculated wheat heads were capped with a plastic bag to maitain humidity for 48 h. After removing the bags, wheat plants were cultured for another 12 days before examination for disease symptoms. The disease index for each strain was estimated by counting diseased spikelets per wheat head at 14 dpi. The inoculated kernels with typical disease symptoms were harvested and assayed for DON production as described [60]. Infection assays with corn silks of cultivar Pioneer 2375 were conducted as described [18]. The extent of discoloration on infected corn silks was measured after incubation at 25°C in a moisture chamber for 5 days [18].

### Assays for conidium morphology and size

Conidia were harvested from 5-day-old CMC cultures as described [22] and examined by DIC microscopy. The ones that lacked foot cells or had fewer than four septa were considered to be defective in conidium morphology. For both PH-1 and the *Fgsch9* mutant, conidium morphology assays were repeated three times and 400 conidia were examined in each replicate. To compare their differences in conidium size, the length and width of conidia of PH-1 and the *Fgsch9* mutant were measured in three independent experiments with at least 80 conidia measured per replicate. The average number of septa in conidia was calculated with data from three experiments of counting septa in 150 conidia per replicate. The resulting data were subjected to analyses of variance (ANOVA) with the SPSS17.0 statistics software package (SPSS Inc., Chicago, IL). Comparisons of the means were analyzed with the protected Fisher’s Least Significant Difference (LSD) test (P = 0.05).

### Targeted deletion of the *MoSCH9* gene in *M. oryzae*

To delete the *MoSCH9* gene, its upstream and downstream flanking sequences were amplified with primer pairs MoSCH9-1F/MoSCH9-2R and MoSCH9-3F/MoSCH9-4R, digested with *Fes*I and *Asc*I, respectively. The resulting PCR products were digested and ligated with the hygromycin resistance cassette (*hph*) released from pCX63 as described [34]. The gene replacement construct was amplified with nest primers MoSCH9-1F’/MoSCH9-4R’ (Table S1) and directly transformed into protoplasts of Ku80. Hygromycin-resistant transformants were screened by PCR with primers MoSCH9-NF/MoSCH9-NR (Table S1) and confirmed by Southern blot analysis.

### Appressorium formation and plant infection with the Δ*Mosch9* mutant

Conidia harvested from 10-day-old oatmeal agar cultures were re-suspended to 10^5^ conidia/ml in distilled water for appressorium formation assays on plastic coverslips as described [61], [62]. Two-week-old seedlings of rice cultivar CO-39 were used for infection assays with conidia re-suspended 0.25% gelation as described [63]. Disease symptoms were observed 7 dpi.

## Supporting Information

Fig. S1 Generation and Southern blot analysis of the Δ*Fgsch9* mutant. **A**. The genomic region of the *FgSCH9* gene and PCR fragments used for generation of the gene replacement construct and Southern blot hybridization. PCR primers were marked with small arrows. E, *EcoR*I. **B**. Southern blots of *EcoR*I-digested genomic DNA of the wild-type strain (PH-1) and Δ*Fgsch9* mutant (SD1) were hybridized with an *FgSCH9* (probe 1) or *hph* fragment (probe 2). Whereas probe 1 hybridized to a 4.4-kb band in PH-1 but not in SD1, probe 2 detected a 2.8-kb band in SD1 that was diagnostic of the gene replacement event.
(TIF)

Table S1 Polymerase chain reaction primers used in this study.
(DOC)
