# Supplementary figures and images for: The Sch9 Kinase Regulates Conidium Size, Stress Responses, and Pathogenesis in Fusarium graminearum
Source: PLoS One. 2014 Aug 21;9(8):e105811. doi: 10.1371/journal.pone.0105811 (PMC4140829; doi:10.1371/journal.pone.0105811)

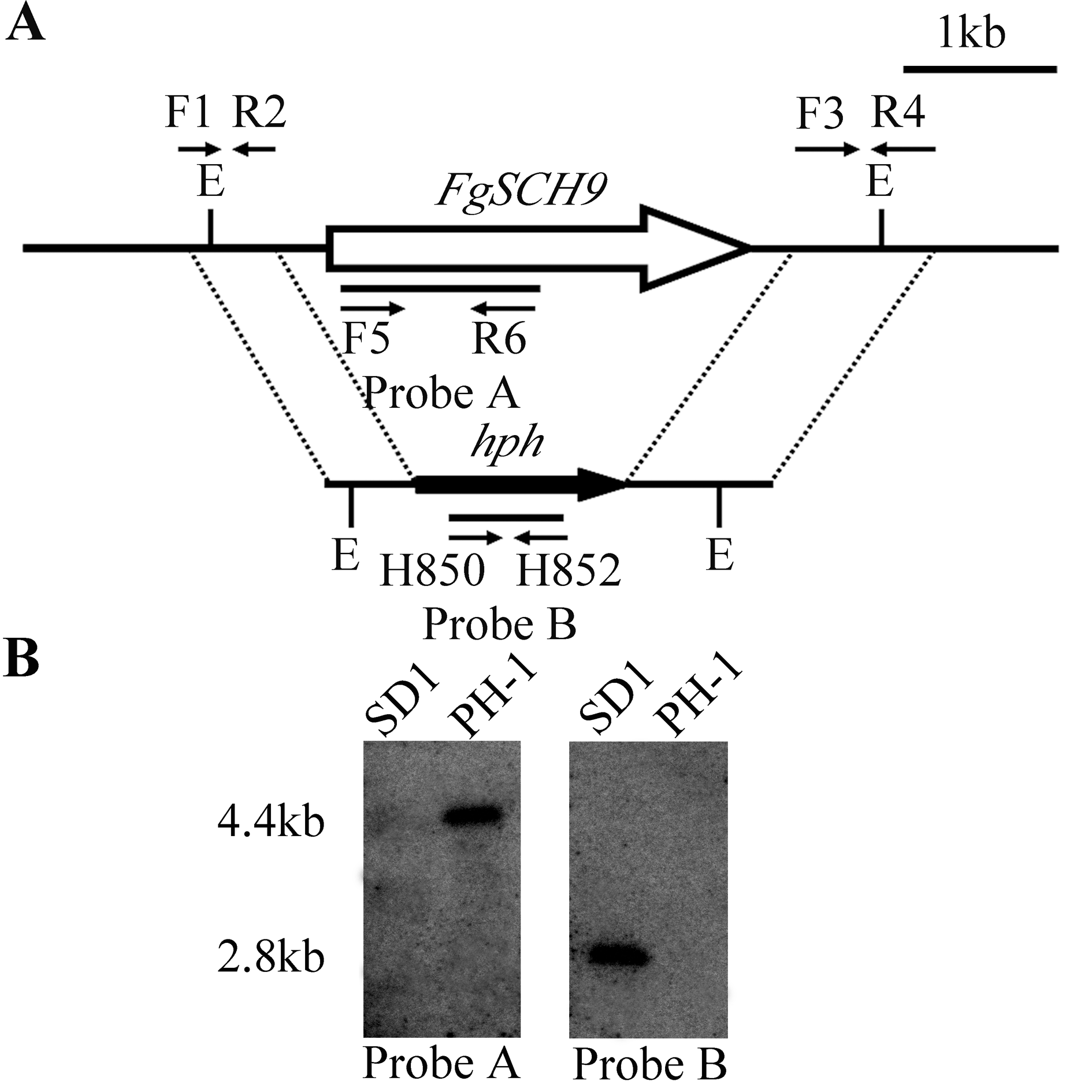

Supplement: Fig. S1 — Generation and Southern blot analysis of the ΔFgsch9 mutant. A. The genomic region of the FgSCH9 gene and PCR fragments used for generation of the gene replacement construct and Southern blot hybridization. PCR primers were marked with small arrows. E, EcoRI. B. Southern blots of EcoRI-digested genomic DNA of the wild-type strain (PH-1) and ΔFgsch9 mutant (SD1) were hybridized with an FgSCH9 (probe 1) or hph fragment (probe 2). Whereas probe 1 hybridized to a 4.4-kb band in PH-1 but not in SD1, probe 2 detected a 2.8-kb band in SD1 that was diagnostic of the gene replacement event. (TIF) [file pone.0105811.s001.tif]
