# Supplementary material for: The Sch9 Kinase Regulates Conidium Size, Stress Responses, and Pathogenesis in Fusarium graminearum
Source: PLoS One. 2014 Aug 21;9(8):e105811. doi: 10.1371/journal.pone.0105811 (PMC4140829; doi:10.1371/journal.pone.0105811)
Supplement: Table S1 — Polymerase chain reaction primers used in this study. (DOC) [file pone.0105811.s002.doc]

**Table S1. Polymerase chain reaction primers used in this study**

| Primer | Sequence (5’-3’) |
| --- | --- |
| F1 | ACGAACAGACCAACGAGCAAACCG |
| R2 | TGACCTCCACTAGCTCCAGCCAAGCCTGGACTCTGGCGATGCGACTATGA |
| F3 | GAATAGAGTAGATGCCGACCGCGGGTTGCATGTGGGCTATGGATGTCTAAT |
| R4 | AAAGAGGGAGGGCTGACACTAAAA |
| F5 | ACCCGATCTACAGGACAAACGC |
| R6 | CCAACCCTCAACAGGAGTGCC |
| F7 | TACGACACCGCCTACTGA |
| R8 | GTCAGGACACCTCCGCTC |
| H850 | TTCCTCCCTTTATTTCAGATTCAA |
| H852 | ATGTTGGCGACCTCGTATTGG |
| H855R | GCTGATCTGACCAGTTGC |
| H856F | GTCGATGCGACGCAATCGT |
| HY/R | GTATTGACCGATTCCTTGCGGTCCGAA |
| YG/F | GATGTAGGAGGGCGTGGATATGTCCT |
| HYG/F | GGCTTGGCTGGAGCTAGTGGAGGTCAA |
| HYG/R | AACCCGCGGTCGGCATCTACTCTATTC |
| COMP/F | CGGGGTACCCTGTTCTACGACACCGCCTACTG |
| COMP/R | AAGGAAAAAAGCGGCCGCCCGACTTTGTTCCCTCCT |
| GFPNAT/F | CGACTCACTATAGGGCGAATTGGGTACTCAAATTGGCATCCTTTCCTCCCCTTCCGTC |
| GFPNAT/R | CACCACCCCGGTGAACAGCTCCTCGCCCTTGCTCACTACGTCAAAATGAGAACCTCCCACC |
| GFP27/F | CAGATCTTGGCTTTCGTAGGAACCCAATCTTCAATGAATGGGGTTATTAACCAAAACGTCAGG |
| MoSCH9-1F | (GCC TGC ATT GCT TGC CTG TCT GTC TTA TG |
| MoSCH9-2R | ATA TGG CCG GCC CAG AAG GCA ATA TTC TTG GGC TGT |
| MoSCH9-3F | AAT TGG CGC GCC GAG TTT GCC AGA GGG TGT ATG GAG |
| MoSCH9-4R | ATC AAT GTT TCT CCG CTG ATG CTC GGT |
| MoSCH9-1F’ | GTC TTA TGG TAT TTC TCC GTC TGC CCG |
| MoSCH9-4R’ | CGC TTG AAC GTC GTT CCC TCA TAA TCA |
| MoSCH9-NF | GTC TTA TGG TAT TTC TCC GTC TGC CCG |
| MoSCH9-NR | CGC TTG AAC GTC GTT CCC TCA TAA TCA |
